# Impact of dengue-preventive behaviors on *Aedes* immature production in Bang Kachao, Samut Prakan Province, Thailand: a cross-sectional study

**DOI:** 10.1186/s12889-020-8394-5

**Published:** 2020-06-11

**Authors:** Pathavee Waewwab, Sungsit Sungvornyothin, Rutcharin Potiwat, Kamolnetr Okanurak

**Affiliations:** 1 Department of Medical Entomology, Faculty of Tropical Medicine, Mahidol University, Bangkok, 10400 Thailand; 2 Department of Social and Environmental Medicine, Faculty of Tropical Medicine, Mahidol University, Bangkok, 10400 Thailand

**Keywords:** Dengue prevention, Behavior, *Aedes* immature production, Vector control

## Abstract

**Background:**

Controlling sites where mosquitos breed is a key strategy in breaking the cycle of infectious transmission of the dengue virus. Preventive behaviors, such as covering water containers with lids and adding temephos (commercially named Abate sand) in water containers are needed to reduce and control mosquito breeding sites. This study aimed to investigate the impact of dengue-preventive behaviors on *Aedes* immature production.

**Methods:**

This cross-sectional study used in-person interviews to record occurrence of dengue-preventive behaviors in Bang Kachao, Samut Prakan Province, Thailand. Larval mosquitos in and around houses were observed and recorded, and covered 208 households.

**Results:**

It was found that 50% of these households had containers for drinking water and 94% used water containers. Covering water containers with effective lids showed the best success among dengue-preventive behaviors for reducing *Aedes* immature production. Adding temephos in water containers also was effective.

**Conclusions:**

Such behaviors substantively affected development of *Aedes* immatures in and around households.

## Background

The World Health Organization (WHO) regards dengue as an emerging and re-emerging mosquito-borne viral disease. Over the last 50 years, dengue has dramatically spread and increased in various locations. Southeast Asia and the Western Pacific in particular have been seriously affected [1]. In Thailand, the first dengue cases were reported in Bangkok in 1949. The first dengue outbreak was in 1958, with 2158 documented cases and 300 deaths [2], The two largest dengue outbreaks in Thailand were in 1997 and 1998, with 101,689 and 126,348 cases, respectively [3, 4]. Dengue is now gradually spreading to rural areas in several parts of Thailand [5].

Dengue is transmitted by *Aedes* spp. mosquitoes, namely *Aedes aegypti* and *Aedes albopictus*, throughout tropical and sub-tropical areas [6]. Female *Aedes* mosquitoes have adapted to improve their chance of life [7], particularly in finding breeding sites for laying eggs in water-holding containers, such as earthen jars used for domestic water storage, flower pots, tires, flower vases, pet water bowls, and disposed of items that fill with rainwater. They can also breed in natural containers, such as tree holes, leaf axils, and coconut shells [8]. The main breeding sites in Thailand were found in earthen jars and in large, rectangular cement containers used to store water for bathing and for flushing toilets [9]. Because of the close relationship between *Aedes* spp. mosquitoes and water-holding containers in/around human houses, vector-control strategies focus on reducing sources of *Aedes* immature (larva and pupa) habitats [10].

As dengue vaccination has limited use, mosquito control remains a vital strategy for preventing and controlling dengue transmission [11]. The WHO noted that water containers without secure lids or tightly fitted mesh screens are potentially the best breeding sites for *Aedes* mosquitoes [12]. Another dengue vector control measure is use of temephos (commercially named Abate sand) as a larvicide. Temephos can be used safely in potable and daily-use water, is recommended by the WHO [13], and is highly effective against *Aedes* larvae [14, 15]. However, its efficacy can be degraded by temperature, organic debris, exposure to sunlight, water use patterns, maintenance of water containers, and refusal to use it in households because of its unpleasant odor [16, 17]. Frequent changing and cleaning of water containers is also effective for considerably reducing *Aedes* larval abundance in households [9, 18]. Additionally, overturning water containers after use, and removing disposed items around the household can help reduce potentially active breeding areas of mosquitoes, thereby reducing *Aedes* immature abundance [19]. Previous studies have revealed many dengue-preventive behaviors that can impact *Aedes* immature production [9, 12, 14, 15, 18–20].

The Ministry of Public Health of Thailand has implemented dengue prevention and control measures called the “3 Do’s”: 1) routinely empty or cover water-storage containers, 2) properly dispose of garbage, and 3) keep houses neat and orderly (Department of Disease Control, Ministry of Public Health of Thailand, 2016).

The present study aimed to investigate the impact of dengue-preventive behaviors on *Aedes* immature production.

## Methods

### Study design and study area

A cross-sectional study was conducted in Bang Kachao, Samut Prakan Province, Thailand (13° 14′ N latitude, 100° 33′ E longitude) during its rainy season (July–September) in 2017. The study areas covered the five villages with the highest dengue incidence rates in Bang Kachao: Ban Hua Ro, Ban Nam Chon, Ban Bang Nam Phueng, Ban Khlong Mon, and Ban Khlong Pae.

### Sample size estimation

The sample size was calculated based on the following formula [21]:

$$ n=\frac{Np\left(1-p\right){Z}^2}{d^2\left(N-1\right)+p\left(1-p\right){Z}^2} $$

where n was the sample size, N was 448 households from the five villages, Z^2^ was the standard 95% confidence interval for a two-sided test (1.96), p was the percentage of households using dengue vector control measures [22], and d^2^ was acceptable completed error (0.05). From the calculation, the study covered 204 households.

### Data collection and data analysis

Field surveys were conducted by three teams, each consisting of two entomologists, a research assistant, and a village heath volunteer. For each interview, the health volunteer first requested permission, then the research assistant interviewed the head of the household, or a representative, by using a structured questionnaire regarding dengue-preventive behaviors. The research objectives and process were explained, and signed, written informed consent to take part in the study was received from each participant. Two entomologists looked for indoor and outdoor water-holding containers and for *Aedes* immatures (larvae and pupae) in such containers. A household found *Aedes* immature production was recorded as “the presence of *Aedes* immature production, and a household with free of *Aedes* immature production was recorded as “the absence of *Aedes* immature production. All *Aedes* immatures were collected in labeled plastic bottles, recorded and transported to the laboratory at the Medical Entomology Insectarium, Faculty of Tropical Medicine, Mahidol University. All *Aedes* immatures were counted. All larvae were identified by species while all pupae were reared to their adult stage before species identification.

Statistical analyses were performed using a statistical program, namely STATA version 14.0 licensed to Mahidol University (Serial number: 401406001858). Descriptive analysis was performed for *Aedes* immatures observed in positive containers and for dengue-preventive behavior in households. The impact of dengue-preventive behaviors on the presence of *Aedes* immature production was analyzed using binary logistic regression, reporting odds ratios. Dengue-preventive behaviors were included as predictors and the presence of *Aedes* immature production as response variables.

## Results

As mentioned, the study covered 208 households in five villages in Bang Kachao. Of these, 105 had drinking water containers (50.48%), and 196 used some form of water container (94.23%). A total of 116 had water vases (55.77%), 103 had pet water bowls (49.51%), and 31 had flower pots (14.90%). There were 145 that washed water containers with a brush or sponge (69.71%), 42 threw away any unused outdoor containers (20.19%), and 71 overturned other containers, such as water buckets or cans, after use (34.13%).

### Aedes immature abundance

A total of 3802 *Aedes* immatures were collected, of which 3725 (97.97%) were *Aedes aegypti* and 77 (2.03%) were *Aedes albopictus*. When observing the proportion of immature mosquito positive container per household, it was found that household with flower pot to be highly infested with Aedes immatures (61%). Households storing used water (15.38%) in bathroom and/or toilet containers, small jars, and large jars were found to be more highly infested with *Aedes* immatures (55%) than were households storing drinking water (2%) (Table 1).

**Table 1:** Proportion of container with *Aedes* immature mosquito per wet container and per household

Type of container	No. of households had containers	No. of wet containers	No. containers with immature mosquito	Proportion of container with immature mosquito per wet container	Proportion of container with immature mosquito per household
Drinking water	105	115	2	0.02	0.02
Used water	208	250	115	0.46	0.55
Flower vase	116	137	5	0.04	0.04
Pet water bowl	103	108	7	0.06	0.07
Flower pot	31	63	19	0.30	0.61
Unused outdoor	208	375	16	0.04	0.08
Water bucket or can	117	132	7	0.05	0.06
Water bowl or glass at cemetery and/or spirit house	111	267	7	0.03	0.06
Tire	21	157	7	0.04	0.33

### Dengue-preventive behaviors

More than 98% of the surveyed households covered their drinking water containers with lids. More than half used water containers with lids and added temephos. About 12% used neither of those methods. Additionally, more than 86% changed water or added temephos in water vases, and 85.43% changed water in pet water bowls. Approximately 74% did not empty water from flower saucers and about 80% did not throw out unused outdoor containers. Of the surveyed households, almost 70% washed their water containers with a brush or sponge. About 54% overturned other containers, such as water buckets or cans, after use (Table 2).

**Table 2:** Dengue preventive behaviors surveyed in households

Variables	No. of inspected households	%
Container for drinking water		
- No lid	2	1.91
- Covered with effective lid	103	98.09
Container for used water		
- No lid or temephos	24	11.54
- Added temephos	37	17.79
- Had effective lid	47	22.59
- Had effective lid and added temephos	100	48.08
Changed water or added temephos in vase		
- No	16	13.79
- Yes	100	86.21
Changed water in pet water bowl		
- No	15	14.56
- Yes	88	85.44
Emptied water from flower pot		
- No	23	74.19
- Yes	8	25.81
Washed water container with brush or sponge		
- No	63	30.29
- Yes	145	69.71
Thrown away unused outdoor containers		
- No	166	79.81
- Yes	42	20.19
Overturned other containers after use		
- No	46	39.32
- Yes	8	6.83
- Yes, and kept away from rain	63	53.85

The households that covered drinking water containers with effective lids showed the best dengue-preventive behavior. Households not covering lids on drinking water were 4 times more likely to have *Aedes* immature stages than those using effective lids (OR = 4.0, *p* value< 0.001). Those not covering lids on used water containers were 3.4 times more likely to have *Aedes* immature stages compared to those using effective lids (OR = 3.4, *p* value< 0.001). While those adding temephos in used water containers were 1.5 times more likely to have *Aedes* immature stages compared to those using effective lids (OR = 1.48, *p* value< 0.001). Households that both covered used water containers with lids and added temephos were 1.7 times more likely to have *Aedes* immature stages compared to those using effective lids (OR = 1.69, *p* value< 0.001). It was also found that the households did not empty water from flower pots were 2.4 times more likely to have *Aedes* immature stages compared to those emptied water (OR = 2.43, *p* value< 0.001). House that did not change water or added temephos in water vases were 1.9 times more likely to have *Aedes* immature stages compared to those that did (OR = 1.85, *p* value< 0.001). It was also found households that did not overturned other container after use or kept away from rain were 1.3 times more likely to have *Aedes* immature compared with those that did (OR = 1.29, p value = 0.001) (Table 3).

**Table 3:** Binary logistic regression analysis of dengue-preventive behaviors affecting the presence of *Aedes* immature production

Variables	Odds Ratios	95% Confidence Interval	*P* value
Container for drinking water (*n* = 105)			
- No lid	4.00	2.94–5.26	<0.001*
- Covered with effective lid (reference)	1.00		
Container for used water covered by lid (*n* = 208)			
- No lid or temephos	3.45	2.63–4.35	<0.001*
- Added temephos	1.48	1.28–1.59	<0.001*
- Had effective lid (Reference)	1.00		
- Had effective lid and added temephos	1.69	1.61–1.75	<0.001*
Changed water or added temephos in vase (*n* = 116)			
- No	1.85	1.69–2.00	<0.001*
- Yes (reference)	1.00		
Changed water in pet water bowl (*n* = 103)			
- No	1.09	1.02–1.17	0.015*
- Yes (reference)	1.00		
Emptied water from flower pot (*n* = 31)			
- No	2.43	2.08–2.77	<0.001*
- Yes (reference)	1.00		
Washed water container with brush or sponge (n = 208)			
- No	1.01	0.92–1.11	0.839
- Yes (reference)	1.00		
Throw away unused outdoor containers (n = 208)			
- No	1.12	1.04–1.23	0.005*
- Yes (reference)	1.00		
Overturned other containers after use (*n* = 117)			
- No	1.29	1.12–1.51	0.001*
- Yes	1.03	0.94–1.18	0.690
- Yes, and kept away from rain (reference)	1.00		

## Discussion

This study showed that covering drinking and used water storage containers with effective lids had a substantial positive impact toward dengue prevention. A previous study had similar findings for household water containers [9]. Adding temephos in used water containers was also found to be an effective dengue-preventive behavior. Previous studies found temephos was effective against *Aedes* mosquito larvae [14, 15]. Additionally, the present study found that both covering lids and adding temephos had a substantial impact on reducing *Aedes* immatures. In line with this, a previous study also found greater efficacy in using a combination of dengue control methods [9].

The present study found that covering water containers with lids was more effective than adding temephos for preventing *Aedes* immature production. The WHO also reported that use of effective lids is a lower-cost vector control than using insecticide [12]. On the other hand, the effectiveness of abate sand may decrease from sunlight that possibly degraded the active compound of temephos [16]. Moreover, households may frequently drain and refill water containers, which could shorten temephos’ residual effectiveness [16]. Additionally, another study found temephos to provide effective control for 2.5–5 (mean, 3) months [23]. The present study also found that cover water containers with lids was superior to both using a lid and adding temephos. This may be because those who took both measures did not perform them both regularly. Distributing free temephos may therefore not justify the necessary time and effort. Based on this, the Ministry of Public Health should change its health prevention strategy from distributing free temephos to subsidizing lids.

Other water containers identified as *Aedes* mosquito breeding sites were flower pots (and their saucers), pet water bowls, water vases, and unused outdoor containers. Emptying water from flower pots was found effective at dengue control. Changing water or adding temephos in water vases and pet water bowls were also effective preventive behaviors. Previous studies reported that avoiding retaining water in such containers was potentially effective at larval control [9, 21]. Another study reported that removal of unused outdoor containers around houses was effective for reducing sources of mosquito breeding sites [18].

Water buckets and cans were other *Aedes* mosquito breeding sites. The present study found it effective to overturn these containers or keep them away from rain. Overturning them decreased how much rainwater they held.

Previous studies reported that washing containers with a brush or sponge to remove mosquito eggs was effective for dengue control [9, 17, 22, 24–26]; however, this study did not find that relationship. This might be because some water containers were large and therefore difficult to wash, especially those that were rectangular, cement, and built in the bathroom corner. Thus, the container’s size may affect the frequency of washing.

This study was limited by collecting data only once, hence the presence of *Aedes* immature production and dengue-preventive behaviors observed in a household cannot represent all seasons. The data collection covered only 208 households, so it might not be sufficient to represent Thailand.

## Conclusions

This study found that covering water containers with effective lids was the most effective method for controlling *Aedes* immature production. Adding temephos to water is also potentially effective. Public health messages should therefore focus on the use of lids, as this appears most successful and costs less than temephos.

## Data Availability

Not applicable.

## Abbreviation

WHO: World Health Organization
